# EFFECTS OF ATTENTIONAL CONTROL DEMANDS IN PROCESSING SPEED TRAINING ACROSS THE ADULT LIFESPAN: FIRST FINDINGS

**DOI:** 10.1093/geroni/igae098.2190

**Published:** 2024-12-31

**Authors:** Claudia von Bastian, Alice Reinhartz, Eleanor Hyde, Shuangke Jiang, Elisabeth Loranger, Jeff Ferreri, Sylvie Belleville, Tilo Strobach

**Affiliations:** University of Sheffield, Sheffield, England, United Kingdom; Medical School Hamburg, Hamburg, Hamburg, Germany; University of Sheffield, Sheffield, England, United Kingdom; University of Sheffield, Sheffield, England, United Kingdom; Institut Universitaire de Geriatrie de Montreal, Montreal, Quebec, Canada; Institut Universitaire De Geriatrie De Montreal, Montreal, Quebec, Canada; University of Montreal, Montreal, Quebec, Canada; Medical School Hamburg, Hamburg, Hamburg, Germany

## Abstract

Evidence for cognitive training-induced far transfer of improvements to untrained cognitive abilities is mixed. One notable exception appears to be training interventions that target speed of processing. However, the mechanisms underpinning these training effects are yet unclear. In this pre-registered, multi-site training study, we tested the hypotheses that (a) training tasks with stronger attentional control demands will induce larger transfer effects, and that (b) gains in the rate of information accumulation (i.e., drift rate) will be positively associated with these effects. For this purpose, we recruited 476 healthy participants spanning the adult lifespan (18-85 years) from three sites in the United Kingdom, Germany, and Canada, who were randomly allocated to one of four groups practising tasks with increasing attentional control demands. Transfer to working memory, executive functions, reasoning, and everyday cognitive functioning was assessed before, immediately after, and 3 months after 10 training sessions. N = 388 participants (age in years M = 48.61, SD = 18.28, range 18 – 85; 218 women, 168 men, 2 participants with undisclosed gender; education in years M = 16.68, SD = 3.68) completed the study. The first results from this study will be presented in this talk.

